# Transcriptome analysis of mycobacteria in sputum samples of pulmonary tuberculosis patients

**DOI:** 10.1371/journal.pone.0173508

**Published:** 2017-03-10

**Authors:** Sumedha Sharma, Michelle B. Ryndak, Ashutosh N. Aggarwal, Rakesh Yadav, Sunil Sethi, Shet Masih, Suman Laal, Indu Verma

**Affiliations:** 1 Department of Biochemistry, Post Graduate Institute of Medical Education and Research, Chandigarh, India; 2 Department of Pathology, New York University Langone Medical Center, New York, New York, United States of America; 3 Department of Pulmonary Medicine, Post Graduate Institute of Medical Education and Research, Chandigarh, India; 4 Department of Medical Microbiology, Post Graduate Institute of Medical Education and Research, Chandigarh, India; 5 Molecular Diagnostic and Research Laboratory (MDRL) Pvt. Ltd., Chandigarh, India; 6 Veterans Affairs New York Harbor Healthcare System, New York, New York, United States of America; Institut de Pharmacologie et de Biologie Structurale, FRANCE

## Abstract

Pulmonary tuberculosis, the disease caused by *Mycobacterium tuberculosis*, still retains a top rank among the deadliest communicable diseases. Sputum expectorated during the disease continues to be a primary diagnostic specimen and also serves as a reservoir of bacteria. The expression pattern of mycobacteria in sputum will lead to an insight into bacterial adaptation at the most highly transmissible stage of infection and can also help in identifying newer diagnostic as well as drug targets. Thus, in the present study, a whole genome microarray of *Mycobacterium tuberculosis* was used to elucidate the transcriptional profile of mycobacteria in the sputum samples of smear positive pulmonary tuberculosis patients. Overall, the mycobacteria in sputum appeared to be in a low energy and low replicative state as compared to *in vitro* grown log phase *M*. *tb* with downregulation of genes involved in ATP synthesis, aerobic respiration and translational machinery. Simultaneously, downregulation was also seen in the genes involved in secretion machinery of mycobacteria along with the downregulation of genes involved in the synthesis of phthiocerol dimycocerosate and phenol glycolipids. In contrast, the majority of the genes which showed an upregulation in sputum mycobacteria were of unknown function. Further identification of these genes may provide new insights into the mycobacterial behavior during this phase of infection and may help in deciphering candidates for development of better diagnostic and drug candidates.

## Introduction

The success of *Mycobacterium tuberculosis* (*M*. *tb*) as a human pathogen is largely due to its ability to switch between latent and active states and to adapt to different environments and stresses. These adaptations are reflected by changes in its global transcriptional profile. The transcriptome profiles of *log* phase *M*. *tb* and stationary phase persister bacteria have been reported to be different [1] and the transcriptome in log phase laboratory *M*. *tb* culture is different from its transcriptome in the lung. It also differs between bacteria isolated from intra-granulomatous regions of the lung compared to bacteria isolated from regions of lung distant from granulomas in *M*. *tb* infected individuals [2]. *M*. *tb* can also distinguish different immune environments as demonstrated in the differences between infection of immunocompetent (BALB/c) and immunocompromised (SCID) mice [3] and during *ex vivo* growth in whole blood from healthy HIV- subjects and from HIV+ patients [4]. Stress-specific transcriptional changes have also been demonstrated *in vitro* during various stress conditions, e.g., exposure to nitric oxide [5,6], hypoxia [7,8], starvation [9], low pH [10] etc. Interestingly, the *M*. *tb* transcriptome during infection of activated macrophages is in many ways a mirror image of the *M*. *tb* transcriptome during infection of alveolar epithelial cells with upregulation of stress- and dormancy-related genes in the former and down-regulation of these in the latter, and upregulation of energy-, replication-, and virulence-related genes in the latter and down-regulation of these in the former [11,12]. This chameleon-like differential gene expression in response to its environment must be considered for identifying new diagnostics, therapeutic and vaccine targets.

Sputum samples are the primary specimen source for diagnosing active pulmonary tuberculosis (PTB), either by acid-fast staining and/or culturing of bacteria or by nucleic acid amplification, e.g., GeneXpert. Active PTB patients are most contagious when they are smear positive for acid-fast bacteria in their sputum and therefore likely to cough up aerosols containing live *M*. *tb*. These mycobacteria in expectorated sputum represent bacteria during the most highly transmissible stage of infection, yet very little is known about the state of the *M*. *tb* transcriptome at this critical time. Garton *et al*. [13] described in their study that bacterial population in this most critical diagnostic sample has a persister phenotype based on the expression patterns of sputum derived *M*. *tb* in their study. Some of the transcripts expressed by *M*. *tb* in sputum may also be used as diagnostic candidates. But, to be used as diagnostic candidates the transcripts should be specifically present in the sputum samples of TB patients and not in the sputum samples from other diseases having similar clinical symptoms. So, to further expand our knowledge of *M*. *tb* gene expression patterns which are highly specific to the PTB disease, we analyzed the expression patterns of RNA isolated from sputum of lung cancer patients to find cross-hybridization of eukaryotic or other microbial transcripts with *M*. *tb* specific oligonucleotides on the array. This study could aid in the identification of highly expressed *M*. *tb* genes in TB patient sputum which have the potential to be exploited as specific diagnostic markers of active TB.

## Materials and methods

### Patient population and sputum collection

Cough-generated (non-induced) sputum was collected from 7 smear positive TB patients before the initiation of therapy and 7 lung cancer patients, in sterile sputum collection vials from the department of Pulmonary Medicine, Post Graduate Institute of Medical Education and Research (PGIMER) Chandigarh, India after taking informed written consent from the patients. Lung cancer sputum samples were taken as disease controls. The study was approved by the Institutional Ethics Committee PGIMER (No.8818/PG11-1TRG/168235). The sputum samples were placed on ice immediately after collection and within an hour of collection.~0.5 ml of sputum was transferred to Primestore Molecular Transport Media (Longhorn Vaccines and Diagnostics) vial already containing 1.5 ml of a proprietary mixture of components meant to kill and lyse *M*. *tb* in the sputum and to retain the integrity of the released nucleic acid [14,15] as per the PrimeStore manufacturer’s instructions. For optimization of RNA isolation techniques from sputum samples in Primestore, *M*. *tb* at two different concentrations (20,000 and 10,000 bacilli) was spiked in Primestore and RNA was extracted using Trizol method. Further to improve the yield of RNA extraction, three different RNA extraction methods namely Trizol, Primextract kit and RNAzol were first optimized for RNA isolation of *M*. *tb* (20,000 bacilli) spiked in Primestore. Of the three methods, extraction with RNAzol resulted in maximum yield (Trizol: 70ng; Primextract: 27.2ng; RNAzol:576ng) and hence was used for extraction of RNA from sputum samples in Primestore.

### RNA isolation

#### a) From log phase broth grown H37Rv *M*. *tb* (Reference RNA)

RNA was isolated from log phase *M*. *tb* culture using Trizol. Bacteria in culture were pelleted by centrifugation and resuspended in 1 ml of TRI reagent (Molecular Research Center) containing 1/100 volume of polyacryl carrier (Molecular Research Center) and transferred into sterile bead beating tubes containing 150 μl of 0.1 mm zirconia beads (Biospec Products, Bartylestville, OK). *M*. *tb* bacilli were lysed by subjecting to bead beater for 3 X 1 min pulses at maximum speed with 2 min rest on ice between the pulses. Samples were transferred to clean tubes and beads in original tube were washed with 100 μl TRI reagent with carrier and added to the previously transferred sample. Samples were incubated at room temperature (RT) for 10 min and centrifuged at 12000Xg for 10 min at 4°C. Supernatant was transferred to clean tubes and 100 μl 1-bromo-3-chloropropane (BCP; Molecular Research Center) was added and shaken vigorously for 15 sec then incubated for 10 min at RT. The mixed phases were centrifuged at 12000Xg for 15 min at 4°C. The upper phase was transferred to a clean tube and 500 μl of isopropanol was added to precipitate RNA. Centrifuged at 12000Xg for 8 min at 4°C. Pellet was washed with 1 ml of 75% ethanol by vortexing for 10 sec and centrifuged at 7500Xg for 5 min at 4°C. The pellet was dried for 3–5 min and resuspended in 170 μl of RNase-free water

#### b) From sputum samples in Primestore

RNA was isolated from sputum samples in Primestore using RNazol (Molecular Research Center). Briefly, 1 ml of Rnazol was added to 400 μl of sputum in Primestore followed by addition of 400 μl of nuclease free water, then incubated for 15 minutes at RT and centrifuged at 12000Xg for 15 minutes. 75% of RNA-containing supernatant was collected in fresh tubes and equal amount of isopropanol was added. After 10 minutes incubation at RT, the RNA precipitate was centrifuged at 12000Xg for 10 minutes. The pellet was then washed twice with 75% ethanol at 4000Xg for 3 minutes and was finally dissolved in 25 μl nuclease free water.

### DNase treatment and RNA purification

To remove DNA contamination, DNase I (Thermo Scientific) and DNase buffer were added to each tube and incubated for 1 hour at 37°C. DNase-treated RNA isolated from cultured *M*. *tb* was purified using the MinElute Clean up kit (Qiagen) and RNA derived from sputum bacteria was purified using RNA Clean up and Concentrator kit (Zymo Research). RNA quantity and quality was determined by nanodrop and Agilent 2100 Bioanalyzer, respectively.

### DNA microarrays (GEO accession number: GSE93316)

For DNA microarrays the DNase treated RNA (50ng) from log phase *Mycobacterium tuberculosis* H37Rv, smear positive and lung cancer samples was amplified, prior to further use in arrays, using the MessageAmp^™^ II-Bacteria RNA Amplification Kit (Ambion^®^). In the amplification procedure RNA was first polyadenylated, reverse transcribed to form double stranded cDNA and *in vitro* transcribed to form antisense RNA. The RNA was then reverse transcribed using superscript III RT and labeled cDNA was synthesized by klenow fragment (Bioprime kit) using Cy3 and Cy5 dyes. [4,16]. The amplified and labeled cDNA was then hybridized to *M*. *tb* arrays obtained from the Center for Applied Genomics (Public Health Research Institute; Newark, NJ). The arrays contained the oligonucleotides which represented all the annotated open reading frames of mycobacteria. Log phase *M*. *tb* H37Rv was used as a reference standard. cDNA from smear positive samples was analyzed in total of 11 chips with 4 samples with dye flip (8 chips) and 3 without dye flip (3 chips). Similarly cDNA from lung cancer samples was analyzed in a total of 9 chips. The microarrays were scanned with Axon 4000B scanner and processed further with GenePix Pro 6.1 software. The data from scanned chips was normalized using the print-tip Lowess method, and the Cy5/Cy3 intensity ratio was determined for each gene [17]. The intensity ratio data obtained from sputum-derived mycobacterial RNA from all subjects in the smear positive and lung cancer groups, with *in vitro* grown *M*. *tb* H37Rv as reference was used to perform Significance Analysis of Microarrays (SAM) with Multiarray Viewer Software on the TMEV website for determination of differentially expressed genes [18]. A two-fold difference (up or down) was considered differentially expressed. Hypergeometric probability and Fisher’s exact test (FET, p-value<0.05) were calculated to identify significantly enriched functional categories among the differentially expressed genes.

### qRT-PCR

Further validation of the microarray results was done using qRT-PCR by looking at the expression of five genes, in the unamplified RNA, isolated from 5 smear positive PTB patients. Primers for carrying out the qRT-PCR were designed using primer3, primer BLAST and primer express softwares [S1 Table]. The RNA isolated from smear positive sputum was subjected to DNase treatment followed by conversion to cDNA using the cDNA kit from Thermo Fisher Scientific according to manufacturer’s instructions. The qRT-PCR was performed using ABI Sybr green mix on the Qiagen real time PCR machine. The mRNA levels of all these genes were normalized to16S ribosomal RNA. The relative gene expression was calculated with respect to *in vitro* grown *M*. *tb*. Statistical analysis was done using Student’s t-test and p<0.05 was taken to be statistically significant.

## Results and discussion

### Gene expression of *M*. *tb* in sputum of smear-positive TB patients

**Fig 1:**
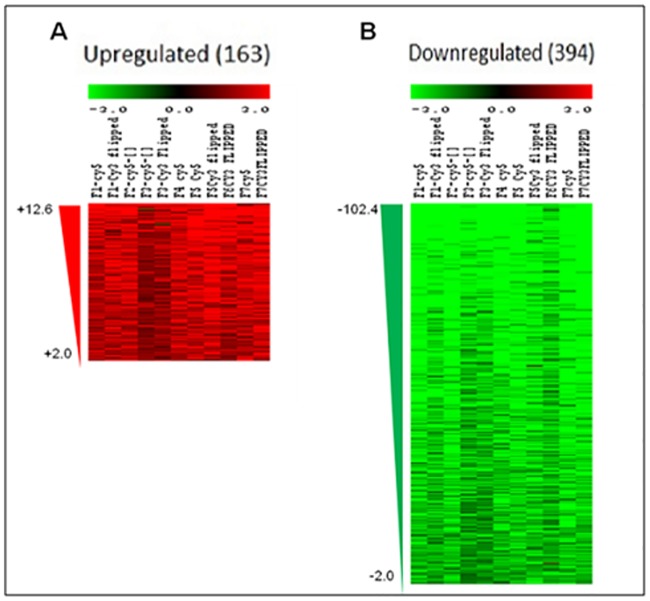
Heat map of the differentially expressed genes in sputum from PTB patients using a mycobacterial whole genome microarray. A) Panel A shows the upregulated genes and B) panel B shows the downregulated genes in sputum from PTB patients as compared to *in vitro* grown *M*.*tb* H37Rv. Red and green coloured triangles represent the amount of fold upregulation or downregulation respectively.

The gene expression profile of *M*. *tb* in smear-positive sputum samples from 7 TB patients was analyzed by *M*. *tb* DNA microarray (4 analyzed with dye flip and 3 analyzed without dye flip for a total of 11 analyzed chips) with RNA from *in vitro* log-phase grown H37Rv *M*. *tb* used as the reference. The microarray chips used in this study each represent the entire annotated *M*. *tb* genome plus 371 predicted open reading frames not annotated at the time of chip design. Though two different methods were used for RNA extraction from reference and test samples, but as per literature, both the methods used for RNA isolation i.e. Trizol and RNAzol have similar basic components and isolating total RNA with RNAzol gives better yield and the same has been observed in the present study also. Mannhalter et al. have reported that although RNA yield was higher in case of RNAzol as compared to Trizol however the expression of ABL gene studied by these authors was comparable using both the methods [19] thus suggesting that different RNA extraction methods do not affect the transcriptional profile of the genes.. Overall, our analysis showed 557 differentially-expressed genes of which 42 are non-annotated (Fig 1; S2 and S3 Tables). In total, 163 genes (24 non-annotated) were upregulated (Fig 1A; S2 Table) and 394 genes (18 non-annotated) were downregulated (Fig 1B; S3 Table). Similar pattern of more downregulated genes as compared to upregulated was also seen in the earlier study done on sputum derived *M*. *tb* [13]. The extensive overall downregulation (almost 2.5 times greater number of genes than upregulated) is noteworthy as microarray studies of *M*. *tb* in macrophages (human non-activated and murine activated) [11,20] and human alveolar epithelial cells [12], which also used *in vitro* log-phase H37Rv RNA as a reference, resulted in 2–3 fold greater numbers of upregulated genes compared to number of downregulated genes. This suggests that the transcriptional patterns of mycobacteria expectorated in the sputum are quite different from the expression patterns of mycobacteria actively growing and replicating in various cell types.

### Functional categorization of differentially expressed genes

The annotated genes which showed a differential expression were sorted as per their functional category designation in the Tuberculist website (http://tuberculist.epfl.ch) (Table 1). Categories of genes that had more members as downregulated compared to upregulated included “Information pathways” (51 down: 2 up), “Cell wall and Cell Processes” (91 down: 19 up), and “Virulence, Detoxification, Adaptation” (21 down: 5 up). These categories are associated with DNA and protein synthesis, cell division, cell wall remodeling, transport across the cell wall, toxin/antitoxin systems, adaptation to stress, and host cell interactions. Number of genes categorized as involved in “Lipid metabolism” or “Intermediary Metabolism & Respiration” observed as downregulated were also more as compared to upregulated (30 down: 10 up and 79 down: 32 up, respectively). The remaining functional categories (Regulatory Proteins, Insertion Sequences & Phages, PE/PPE, as well as Conserved Hypotheticals and Unknowns) showed a less than 2-fold difference between numbers of upregulated and downregulated genes, with PE/PPE the only category having a higher number of upregulated genes (10 down: 14 up). Interestingly, “Conserved Hypotheticals”, “Unknown”, and the non-annotated genes on the chip made upto ~40% of the upregulated genes. Understanding the roles of these genes could expand our knowledge of the state of *M*. *tb* at this stage of infection.

**Table 1:** Functional categories of differentially expressed genes.

Functional Category^a^	Numbers of Annotated Genes Differentially Expressed		
	Upregulated (Hypergeometric probability)	Downregulated (Hypergeometric probability)	Total
Conserved Hypotheticals (1042)^b^	40 (0.061759406)^c^	67 **(8.16275E-07)**	107
Cell Wall and Cell Processes (772)	19 (**0.001995823)**	91 **(0.010574298)**	110
Intermediary Metabolism & Respiration (936)	32 **(0.03347788)**	79 **(0.008638868**	111
Virulence, Detoxification, Adaptation (239)	5 **(0.035212069)**	21 (0.074186339)	26
Lipid Metabolism (272)	10 (0.120666963)	30 (0.068644912)	40
PE/PPE (168)	14 **(0.005487782)**	10 **(0.020306077)**	24
Regulatory Proteins (198)	10 (0.108673767)	18 (0.090307358)	28
Information Pathways (242)	2 **(0.001542606)**	51 **(6.06728E-08)**	53
Insertion Sequences and Phages (147)	6 (0.166936362)	8 **(0.017835861)**	14
Unknown (15)	1 (0.34490959)	1 (0.342707614)	2
Total	139	376	515

^a^ Functional Categories as designated on the Tuberculist website (http://tuberculist.epfl.ch)

^b^ Numbers in parentheses indicate total number of genes in the category in the *M*. *tb* H37Rv genome.

^c^ Number in parentheses indicate hypergeometric probability p value and bold values indicate statistical significance at p<0.05

### Transcriptional profile of *M*. *tb* in sputum indicates a low energy, low replicating, non-aerobic state

Actively replicating *M*. *tb* requires energy and protein synthesis. Of the 8 genes that encode ATP synthase, 7 (*atpA*,*B*,*D*,*E*,*F*,*G*, and *H*) were downregulated (p<0.001), and 6 of the 22 genes encoding 30S (p<0.05) and 16 of the 36 genes encoding 50S (p<0.001) were also downregulated (Table 2,S4 Table). These results are consistent with the results seen in other studies done with the sputum of TB patient [13,21] where the genes encoding ATP synthase have been found to be downregulated along with the downregulation of genes involved in ribosomal function in *M*. *tb* from sputum. *M*. *tb* utilizes an aerobic respiratory pathway during the first few weeks of infection of the mouse lung with oxygen as the final electron acceptor while *M*. *tb* transitioning to a dormant, non-replicating state uses an alternate pathway with nitrogen as the final electron acceptor [22]. The aerobic pathway components include NADH-DH 1 (*nuoA-N*), cytochrome c reductase (*qcrCAB*), and cytochrome c oxidase (*ctaCDE*). The alternative respiratory pathway components include NADH-DH 2 (*ndh or ndhA)* and nitrate reductase (*narGHJI*). The transcriptome of sputum-derived mycobacteria indicates suppression of the aerobic pathway as 5/14 genes encoding NADH-DH 1 (p<0.01, Table 3) are downregulated as are *ctaE* and *qcrA* and *B* (p<0.05; Table 3, S5 Table). None of the genes encoding alternate pathway components were differentially expressed with the exception of *narH*, which was interestingly also downregulated. Thus the expression pattern of these genes in mycobacteria from sputum further points towards a low energy, slow growing state of mycobacteria with downregulation of respiratory pathway as observed by various other groups. [13,21,23]

**Table 2:** Differentially expressed functional categories indicative of *M*. *tb* replication.

Functional Group	Description/Association	# of Genes	Hypergeometric Probability (FET p-value)	Range of Fold Change
30S ribosomal proteins (22)^a^	Protein synthesis	7(down)	0.0035* **(0.0044)** **	-2.4 to -12.8
50S ribosomal proteins (36)	Protein synthesis	16 (down)	**8.777E-08 (7.682E-08)**	-2.1 to -16.1
ATP Synthase (8)	Energy	7 (down)	**7.165E-07 (7.068E-07)**	-2.7 to -8.7

^a^ Number within parentheses indicates total number of genes in the *M*. *tb* genome within this functional group

*Hypergeometric probability p value.

**Fisher’s exact test probability p value and bold numbers indicate statistical significance at p-value ≤ 0.05.

**Table 3:** Differentially expressed functional categories indicative of *M*. *tb* respiratory state.

Functional Group	Description/Association	# of Genes	Hypergeometric Probability (FET p-value)	Range of Fold Change
NADH DH1 (14)^a^	Aerobic respiration	5 (down)	0.0078*(0.0092)**	-2.5 to -9.2
Cytochrome c Reductase (3)	Aerobic respiration	2 (down)	**0.0271 (0.0281)**	-5.2 to -9.1
Cytochrome c Oxidase (4)	Aerobic respiration	1 (down)	0.2925 (0.3452)	-5.9
NADH DH2 (2)	Alternative Electron Transfer	NDE^b^	N/A	N/A
Nitrate Reductase & Transport (5)	Non-aerobic respiration	1 (down)	0.3290 (0.4110)	-2.5

^a^ Number within parentheses indicates total number of genes in the *M*. *tb* genome within this functional group

*Hypergeometric probability p value.

**Fisher’s exact test probability p value and bold numbers indicate statistical significance, at p-value ≤ 0.05.

^b^not differentially expressed

### Downregulation of synthesis pathways for virulence-associated cell wall components (PDIM/PGLs)

Mycobacteria share a unique and complex cell wall that makes the bacteria acid-fast, however, there are also cell wall components that are restricted to and associated with virulent strains. Phthiocerol dimycocerosates (PDIMs) and phenol glycolipids (PGLs), the two closely related components of cell wall, which are surface-exposed, are seen in pathogenic mycobacteria where they have been linked to virulence in animal models [24–26]. The ability to produce PGLs is considered a factor contributing to the hyper-lethality of the Beijing/W strains of *M*. *tb* [27]. In this study, a number of genes involved in cell wall synthesis were downregulated, but most notably were genes involved in phthiocerol dimycocerosate (PDIM) and phenol glycolipid (PGL) synthesis and transport (p<0.01; Table 4, S6 Table); specifically (Rv2930 (*fadD26*), Rv2935 (*ppsE*), Rv2936 (*drrA*), Rv2937 (*drrB*), Rv2941 (*fadD28*), Rv2942 (*mmpL7*), Rv2948c (*fadD22*), Rv2950c (*fadD29*), and Rv2959c, all located within the DIM gene cluster (Rv2928-Rv2962c) of *M*. *tb* [28,29]. The collective downregulation of several genes associated with PDIM/PGL synthesis and transport in sputum-derived *M*. *tb* suggests the suppression of both virulence and host interacting activity as it may not be required at this stage of infection. However it has been seen that after the bacteria infects human alveolar epithelial cells the expression of several of PDIM/PGL is upregulated [12] .The downregualtion of these genes in sputum *M*. *tb* may be attributed to the downregulation of the another gene Rv2048c (pks 12), disruption of which has been associated with. [30] attenuation of virulence in an intra-nasally-infected mouse model [31]. Besides, *pks12* also produces a novel lipid antigen which is presented by CD1c to T cells [32], thus downregulation of *pks12* can be seen as a immune evasion strategy devised by mycobacteria planning to establish a successful infection in new host.

**Table 4:** Virulence-associated cell wall synthesis and transport.

Functional Group	Description/Association	# of Genes	Hypergeometric Probability (FET p-value)	Range of Fold Change
PDIM/PGL Synthesis/transport (36)^a^	Cell wall/ virulence	9 (down)	0.0054* (0.0075)**	-2.7 to -5.4

^a^ Number within parentheses indicates total number of genes in the *M*. *tb* genome within this functional group

*Hypergeometric probability p value.

**Fisher’s exact test probability p value and bold numbers indicate statistical significance at p-value ≤ 0.05

### Downregulation of genes for protein export systems in *M*. *tb*

Protein transport across the *M*. *tb* cell wall can occur via several different secretion systems, the “sec” secretion system, the twin-arginine translocation “tat” secretion system as well as several specialized “type VII” secretion systems encoded by similarly composed but distinct genomic loci designated “ESX 1-ESX-5” which may have resulted from a series of duplication events and each ESX secretion system appears to have a divergent function. In this study, 3 genes (Rv1440, Rv2586c, Rv2587c) of 8 contributing to sec-dependent secretion (p<0.05) and 1 gene (Rv2094c) of 4 comprising the tat pathway were downregulated in sputum-derived bacteria (Table 5, S7 Table). Additionally, multiple genes located in ESX-1, ESX-3, and ESX-5 loci were also downregulated. Specifically, in the ESX-1 locus of 20 genes, Rv3871, Rv3872, Rv3875 (*esat-6*, *esxA*), Rv3874 (*cfp-10*, *esxB*), and Rv3878 were downregulated (p<0.05); in the ESX-3 locus of 11 genes, Rv0282, Rv0284, Rv0286, Rv0288 (*esxH*), and Rv02920 were downregulated (p<0.01); and in the ESX-5 locus of 17 genes, Rv1783, Rv1792 (*esxM*), Rv1793 (*esxN*), and Rv1795 were downregulated (Table 6, S8 Table). The downregulation of these genes suggests decreased protein export by *M*. *tb* in TB patient sputum. Therefore along with decreased synthesis of proteins in sputum *M*. *tb* decreased export of proteins further points towards a low metabolic state of *M*. *tb* in sputum from PTB patients.

**Table 5:** Differentially expressed genes of *M*. *tb* sec and tat secretory pathways.

Functional Group	Description/Association	# of Genes	Hypergeometric Probability (FET p-value)	Range of Fold Change
Sec pathway (8)^a^	Protein secretion	3 (down)	0.0332* (0.0383)**	-2.8 to -3.4
Tat pathway (4)	Protein secretion	1 (down)	0.2925 (0.3452)	-2.6

^a^ Number within parentheses indicates total number of genes in the *M*. *tb* genome within this functional group

*Hypergeometric probability p value.

**Fisher’s exact test probability p value and bold numbers indicate statistical significance at p-value ≤ 0.05

**Table 6:** Differentially expressed genes located within ESX type VII secretion system loci.

Functional Group	Description/Association	# of Genes	Hypergeometric Probability (FET p -value)	Range of Fold Change
ESX-1 (20)^a^	ESAT-6 secretion[33]	5 (down)	0.0321*(0.0434)**	-2.1 to -8.3
ESX-2 (12)	Unknown	NDE^b^	NA	NA
ESX-3 (11)	Iron/Zinc homeostasis[33,34]	5 (down)	**0.0024 (0.0028)**	-2.5 to -4.9
ESX-4 (7)	Most ancestral [35]	NDE	NA	NA
ESX-5 (17)	PE/PPE secretion [36–38]	4 (down)	0.0610 (0.0832)	-2.8 to -10.4

^a^ Number within parentheses indicates total number of genes in the *M*. *tb* genome within this functional group

*Hypergeometric probability p value.

**Fisher’s exact test probability p value and bold numbers indicates statistical significance at p-value ≤ 0.05

^b^Not defined

### Downregulation of genes encoding ESAT-6 and ESAT-6-like proteins

The expression of the genes for ESX-1-encoded and secreted proteins ESAT-6 and CFP-10 was found to be downregulated (8-fold and 4-fold, respectively) in this study. ESAT-6 is a virulence factor that has cytolytic activity and contributes to dissemination from the lung in animal models [39–41]. Mycobacterial *cfp-10* encodes a protein of similar structure that binds to and is co-secreted with ESAT-6 [42,43]. A ~23 member family of proteins in *M*. *tb*, including ESAT-6 and CFP-10, have been defined as “ESAT-6-like” according to their small size (~100 amino acid length), predicted helix-turn-helix arrangement as well as the central WXG motif [44,45]. In addition to *esat-6* and *cfp-10*, 9 other genes encoding ESAT-6-like proteins were also downregulated (p<0.01). Among these, *esxH* is located in the ESX-3 locus, and *esxM* and *esxN* are located in the ESX-5 locus. The other genes encoding ESAT-6-like proteins which were downregulated i.e., *esxI*, *esxJ*, *esxK*, *esxP*, *esxV* and *esxW* (p<0.001) are located outside ESX loci (Table 7, S9 Table). While the functions of these genes are not known, the shared structures could indicate similar activities. Interestingly, the ESX-1 and ESX-5 loci and *esat-6*, *cfp-10*, *esxJ*, *esxK*, and *esxP*, (and additionally *esxL* and *esxW*) are reported to be upregulated during replication of *M*. *tb ex vivo* in whole blood from HIV+ patients [4]. and in *M*. *tb* infected type II alveolar epithelial cells [12]. Thus as the high expression of these genes is required when *M*. *tb* encounters a new host, but is not present in sputum *M*. *tb*, this further indicates that the sputum bacteria are in a transition phase.

**Table 7:** Differentially expressed genes encoding ESAT-6-like proteins.

Functional Group	Description/Association	# of Genes	Hypergeometric Probability (FET p-value)	Range of Fold Change
Total ESAT-6-like in genome (23)^a^	Similar structure to ESAT-6	11 (down)	4.044E-06* (3.612E-06)**	-3.2 to -18.3
ESAT-6-like Outside ESX loci (13)	Do not reside in ESX loci	6 (down)	**0.0008 (0.0009)**	-3.2 to -18.3

^a^ Number within parentheses indicates total number of genes in the *M*. *tb* genome within this functional group

*Hypergeometric probability p value.

**Fisher’s exact test probability p value and bold numbers indicate statistical significance at p-value ≤ 0.05

### Differential expression of two-component response regulators MprA and PhoP

Two-component systems (TCS), which are composed of paired sensor histidine kinases and DNA-binding response regulators, serve as mechanisms enabling the bacteria to respond and adapt to specific environmental conditions by altering the expression of a specific set of genes Interestingly, transcripts for two two-component response regulators; *mprA* and *phoP* were found to be differentially expressed by *M*. *tb* in sputum from smear-positive TB patients; *mprA* was upregulated, and *phoP* was downregulated. MprA is a part of two component system MprAB and is required for the bacteria to adapt during persistent infection [46] and to respond to stress conditions [47] by regulating the expression of several downstream genes [48], In the current study downregulation of *atp*, *nuo* and *espA* genes in TB sputum may be a downstream effect of *mprA* upregulation. *espA* operon is a gene cluster essential for proper functioning of ESX-1 secretion system and its promoter site has binding region for MprA [49] Along with MprA, regulation of *espA* operon is also done by another two component system PhoPR which upregulates its expression [49–51]. It is an important system which regulates bacterial virulence [47] by regulating the expression of more than hundred proteins including cell wall components and mycobacterial ESX-1 secretion system [47], *nuo* genes [52]; lipid metabolism and complex lipid biosynthesis genes, genes within the ESX-1 locus [52]. The expression of most of these genes has shown a concordance with *phoP* expression in the current study. Also another gene *whiB6* which is known to be overexpressed in *phoP* mutant [50], has been seen as upregulated in the current study. Further, consistent with the *phoP* downregulation, six other genes were found to be downregulated in TB sputum samples (Rv1639c, Rv2376c, Rv2391, Rv2396, Rv3477, and Rv3479) which have also been found to be downregulated in a *phoP* mutant compared to wild type (WT) *M*. *tb* [50]. Thus, the coordinated upregulation of *mprA* and downregulation of *phoP* is consistent with our other results described above.

### Transcriptional profile of sputum *M*. *tb* distinct from dormancy

Interestingly, despite the downregulation of genes involved in energy production, aerobic respiration, and bacterial replication, which is usually associated with a stress response leading to transition to dormancy and is correlated with the activity of the Dev(Dos)RS two-component system, neither *dosR* nor *dosS* were differentially expressed by bacteria in sputum. In fact, of the 48 genes in the DosR regulon, 6 were found to be downregulated. Eight genes of the DosR regulon have earlier been shown to be significantly higher expressed in WT *M*. *tb as* compared to its *phoP* mutant [48], thus suggesting cross-talk between the DosRS and PhoPR two-component systems, with PhoP inducing the DosR regulon, at least in part. The downregulation of *phoP* may be responsible for the downregulation of some genes in this regulon in the current study. Thus, as the dormancy regulon is not upregulated even though the *M*. *tb* in sputum is showing the transcriptional profile similar to stress response, with an upregulation of *mprA* involved in *M*. *tb* persistence, it can be suggested that the bacteria is in a phase of persistence infection.

### Upregulated genes as diagnostic candidates

**Fig 2:**
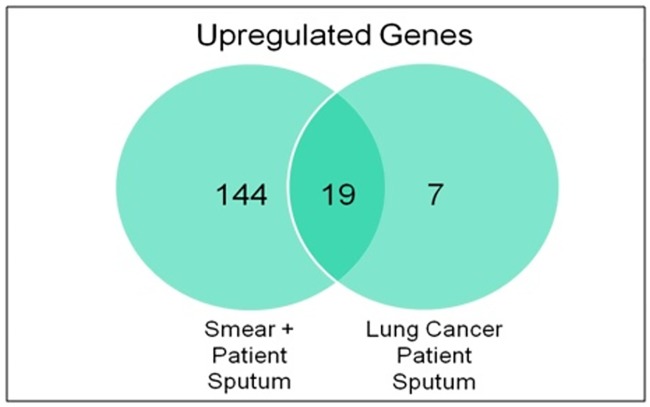
Venn diagram indicating overlap of *M*. *tb* genes detected as upregulated in sputum in TB patients and in sputum from lung cancer patients. 163 genes were upregulated in sputum from PTB patients and 26 genes also showed upregulation in sputum from lung cancer patients out of which 19 genes were common between the two and hence were eliminated from the list of diagnostic candidates

The upregulated genes in the TB patient sputum, though less in number, but represent the transcriptional requirement of mycobacteria at that stage of human infection. They can be further used as disease specific biomarkers in sequence based diagnostic tests after assessment of any cross-reactivity with the transcripts expressed by eukaryotes or any other microbiome genes. Therefore the expression of the mycobacterial genes was also assessed in RNA isolated from lung cancer patient sputum (n = 7) which were confirmed *M*. *tb* culture negative. Unexpectedly, 26 *M*. *tb* genes showed a statistically significant increased signal (>2fold) by microarray analysis of lung cancer sputum bacterial RNA when compared to the reference *in vitro* grown *M*. *tb* RNA. The increased signal for mycobacterial genes in lung cancer patients can be either due to cross-reactivity of these transcripts with other eukaryotic or microbiome transcripts (i.e. non specific binding) or due to presence of latent bacilli in these patients. However, it has been reported that a non-target transcript can result in a significant signal only if there is >75% similarity with the target sequence [53]. BLAST analysis of oligonucleotides on the microarray corresponding to these 26 upregulated genes in lung cancer did not result in any cross-reactivity with eukaryote/microbiome related sequences. Thus, the alternative possibility for the current expression pattern can be the presence of latent TB disease as they are from a TB endemic country. Earlier it has been reported that more than one quarter of lung cancer patients from a TB endemic country were having latent TB infection [54]. Moreover, in a study carried out by Barrios-Payán *et al*. latent bacilli have been seen in the autopsy samples of non-TB patients from various cell types including pneumocytes, endothelial cells and macrophages [55]. So, the latent *M*. *tb* bacteria residing in the alveolar cells might get expectorated by the lung cancer patients rendering some degree of hybridization with the microarray chips. Out of these 26 genes 19 genes were overlapping with the upregulated genes in TB patient sputum (Fig 2, S10 Table). As these genes does not specifically represent the transcriptional profile of mycobacteria during active stage of infection, they should be excluded as potential markers for active TB. Among the rest of the genes (n = 143, Fig 2), which are specifically upregulated in active TB patients further selection can be made based on two criteria, either the selection of genes with highest expression or the selection of genes which are present exclusively in mycobacteria and absent even from the vaccine strain BCG. One such group of genes is the region of difference (RD) genes of mycobacteria, some of which have also shown upregulation in the current study. Overall, 6 RD genes have been found to be > 2 fold or nearly 2 fold upregulated in the current study. The upregulated RD genes involve Rv2351c from RD5, Rv1965 and Rv1971 from RD7, Rv2657c and Rv2674 from RD11 and Rv3121 from RD12. As these genes have been found to be specifically upregulated in PTB sputum mycobacteria, these genes and their corresponding proteins can be further explored for their diagnostic potential in molecular and immunological assays respectively.

### qRT-PCR validation

**Fig 3:**
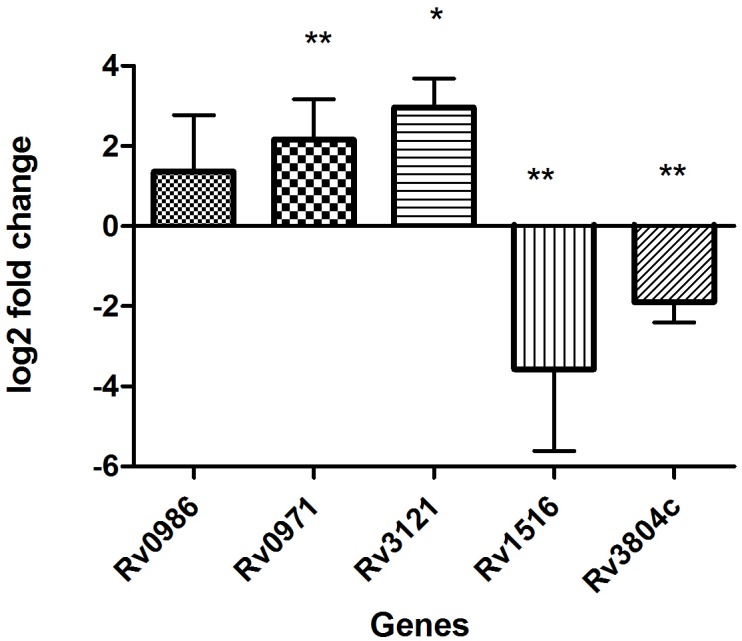
Validation of microarray data on real time PCR by analyzing the relative expression of 5 genes in smear positive PTB samples as compared to *in vitro* grown *M*.*tb* H37Rv. 16s rRNA was used as reference gene for normalization. Y-axis values (Log2 fold change) of ≥1 indicate upregulation and values ≤ -1 indicate down-regulation. Each bar represents mean ± SD values for each of the genes with three technical replicates. *p<0.05; **p<0.01 by student’s t test.

The results of microarray were also validated using qRTPCR (Fig 3). Five genes were used for validation which includes the topmost expressed Rv0986, Rv0971 along with one RD genes Rv3121 and two randomly selected genes; Rv1516 and Rv3804c (*ag85A*). All the validation work was done on unamplified RNA from the same samples that were used for microarray. Similar to the microarray both Rv0986 & Rv0971 along with the RD gene Rv3121 showed upregulation in qRT-PCR, though the upregulation in Rv0986 was not statistically significant. Amid the randomly selected genes Rv3804c showed a significant downregulation in accordance to microarray, but the other gene Rv1516 which was not differentially expressed in microarray showed a downregulation in qRT-PCR. Thus, out of the 5 genes selected for validation on qRT-PCR, the results for 4 genes showed concordance with microarray results and only one gene showed different results.

### Concluding remarks

Sputum mycobacteria from PTB patients appears to be in a low energy, low replicative, low metabolic phase with decreased virulence and protein export. The apparent low replicative/metabolic state of *M*. *tb* in sputum is distinct from transcriptional patterns associated with dormancy as neither dosR nor dosS genes are differentially expressed. Thus, we propose that these bacteria are in a unique metabolic state in order to conserve resources as it prepares to enter the external environment prior to infecting a new host. Upon entering the new host, contact with the respiratory environment may act as a trigger for transcriptional adaptations that promote infection. Also, considering the upregulation of a number of unknown genes for this unique adaptation of mycobacteria in sputum before infecting a new host, further characterization of the (unknown/conserved hypotheticals/non-annotated) genes may help to expand nucleic acid amplification/sputum-based TB diagnostics, and the future understanding of the as yet unknown functions/roles of these genes at this stage of infection.

## Supporting information

S1 Table List of primers used in qRT-PCR. (DOCX)

S2 Table Upregulated mycobacterial genes in sputum of smear positive patients. (DOCX)

S3 Table Downregulated mycobacterial genes in sputum of smear positive patients. (DOCX)

S4 Table Identity of differentially expressed functional categories genes indicative of *M*. *tb*. (DOCX)

S5 Table Identity of differentially expressed functional category genes indicative of *M*. *tb* respiratory state. (DOCX)

S6 Table Identity of differentially expressed virulence-associated cell wall synthesis and transport genes. (DOCX)

S7 Table Identity of differentially expressed genes of *M*. *tb* sec and tat secretory pathways. (DOCX)

S8 Table Identity of differentially expressed genes located within ESX type VII secretion system loci. (DOCX)

S9 Table Identity of differentially expressed genes encoding ESAT-6-like proteins. (DOCX)

S10 Table Upregulated genes in lung cancer. (DOCX)
