# Supplementary material for: Transcriptome analysis of mycobacteria in sputum samples of pulmonary tuberculosis patients
Source: PLoS One. 2017 Mar 10;12(3):e0173508. doi: 10.1371/journal.pone.0173508 (PMC5345810; doi:10.1371/journal.pone.0173508)
Supplement: S1 Table — (DOCX) [file pone.0173508.s001.docx]

| **Gene Name** | **Primers** |
| --- | --- |
| Rv3804c | F: 5’-TGGCCAGTCAAGCTTCTACT-3’  R: 5’-CGAAGAAGCAGCCATCGAAA-3’ |
| Rv3121 | F: 5’-AATTCCTGGACCCCGATGAG -3’  R: 5’-CAAACGGTCTTGCGAGTTGA-3’ |
| Rv1516 | F:5’-TCATCAAGATCAACGCATCCA-3’  R: 5’-CCGCCAAGCAGGTAAAACC-3’ |
| Rv0986 | F: 5’-TTCCCGATAAACTCTCCGGC-3’  R: 5’-GGCTAACACCAGCATGGGAT-3’ |
| Rv0971 | F:5’-CCAGGCTGACACTGAACTCT-3’  R’:5’-TATGCCCCAGCACGACCA-3’ |
| 16S | F: 5’- TTGTCTCATGTTGCCAGCAC-3’  R: 5’- ACCGGCTTTTAAGGATTCGC-3’ |

**S1 Table: List of primers used in qRT-PCR**
