# Supplementary material for: Transcriptome analysis of mycobacteria in sputum samples of pulmonary tuberculosis patients
Source: PLoS One. 2017 Mar 10;12(3):e0173508. doi: 10.1371/journal.pone.0173508 (PMC5345810; doi:10.1371/journal.pone.0173508)
Supplement: S2 Table — (DOCX) [file pone.0173508.s002.docx]

**S2 Table: Upregulated mycobacterial genes in sputum of smear positive patients**

| **Name** | **ID** | **Fold Change** |
| --- | --- | --- |
| Rv0986 | Rv0986 | 12.62094 |
| echA7 | Rv0971c | 9.115335 |
| Rv0093c | Rv0093c | 7.823367 |
| Rv1192 | Rv1192 | 5.205209 |
| hypothetical protein | ORF03310 | 5.129227 |
| hypothetical protein | ORFD0025 | 4.685376 |
| hypothetical protein | ORF00830 | 4.629749 |
| Rv1582c | Rv1582c | 4.621984 |
| Rv2843 | Rv2843 | 4.458276 |
| hypothetical protein | ORF01974 | 4.438266 |
| ephB | Rv1938 | 4.432979 |
| transposase, putative FRAMESHIFT | ORF06256 | 4.327914 |
| Rv2416c | Rv2416c | 4.206878 |
| Rv0397 | Rv0397 | 3.758046 |
| PE_PGRS family protein | ORFD0070 | 3.682038 |
| hypothetical protein | ORF03620 | 3.656207 |
| Rv2541 | Rv2541 | 3.620408 |
| Rv3572 | Rv3572 | 3.508976 |
| Rv0981 | Rv0981 | 3.472372 |
| Rv0095c | Rv0095c | 3.468489 |
| Rv1961 | Rv1961 | 3.388004 |
| Rv0366c | Rv0366c | 3.357285 |
| Rv2980 | Rv2980 | 3.334562 |
| Rv0387c | Rv0387c | 3.323413 |
| PE_PGRS family protein | ORF04443 | 3.31472 |
| fadB5 | Rv1912c | 3.312054 |
| hypothetical protein | ORFD0105 | 3.308936 |
| PE | Rv3539 | 3.268511 |
| Cdd | Rv3315c | 3.242767 |
| mscL | Rv0985c | 3.183177 |
| PPE | Rv3018c | 3.148411 |
| galT' | Rv0618 | 3.144668 |
| Rv2423 | Rv2423 | 3.121817 |
| Rv0331 | Rv0331 | 3.074653 |
| Rv0566c | Rv0566c | 3.064416 |
| conserved hypothetical protein | ORF01163 | 3.048959 |
| rocA | Rv1187 | 3.031168 |
| Rv0837c | Rv0837c | 3.021219 |
| hypothetical protein | ORF06004 | 3.010968 |
| Rv0789c | Rv0789c | 3.005001 |
| Rv1170 | Rv1170 | 2.995586 |
| Rv0294 | Rv0294 | 2.974694 |
| PE_PGRS | Rv0279c | 2.968075 |
| Rv1112 | Rv1112 | 2.96424 |
| Rv0376c | Rv0376c | 2.937025 |
| udgA | Rv0322 | 2.924057 |
| Rv2179c | Rv2179c | 2.918853 |
| Rv2432c | Rv2432c | 2.910874 |
| hypothetical protein | ORF04119 | 2.887237 |
| Rv0308 | Rv0308 | 2.873026 |
| Rv1675c | Rv1675c | 2.849219 |
| Rv1140 | Rv1140 | 2.845272 |
| Cobs | Rv2208 | 2.816609 |
| Rv3845 | Rv3845 | 2.77561 |
| cysN | Rv1286 | 2.767159 |
| ligA | Rv3014c | 2.750006 |
| PE family protein | ORFD0295 | 2.741642 |
| PPE | Rv3347c | 2.728101 |
| Rv2422 | Rv2422 | 2.717344 |
| Rv3836 | Rv3836 | 2.700714 |
| nagA | Rv3332 | 2.694082 |
| fadE7 | Rv0400c | 2.690408 |
| Rv1090 | Rv1090 | 2.690375 |
| Rv0192 | Rv0192 | 2.681422 |
| lpqJ | Rv0344c | 2.667203 |
| Rv1434 | Rv1434 | 2.66418 |
| Rv0836c | Rv0836c | 2.650348 |
| rocD1 | Rv2322c | 2.647374 |
| Rv3706c | Rv3706c | 2.620302 |
| gabD2 | Rv0234c | 2.604694 |
| Rv1674c | Rv1674c | 2.559997 |
| echA13 | Rv1935c | 2.553828 |
| Rv2816c | Rv2816c | 2.548316 |
| Rv0601c | Rv0601c | 2.546502 |
| hypothetical protein | ORF01546 | 2.544128 |
| Rv1190 | Rv1190 | 2.5362 |
| Rv2804c | Rv2804c | 2.526088 |
| PE | Rv0152c | 2.523748 |
| Rv0648 | Rv0648 | 2.50596 |
| Rv0098 | Rv0098 | 2.497207 |
| Sera | Rv2996c | 2.482135 |
| Rv0523c | Rv0523c | 2.454745 |
| Ppa | Rv3628 | 2.450884 |
| Gca | Rv0112 | 2.450867 |
| ureG | Rv1852 | 2.448021 |
| PE | Rv2340c | 2.427163 |
| Rv0498 | Rv0498 | 2.412687 |
| Rv3238c | Rv3238c | 2.385782 |
| molybdopterin oxidoreductase | ORF00812 | 2.383716 |
| PE_PGRS family protein | ORFD0022 | 2.366497 |
| Rv3591c | Rv3591c | 2.360344 |
| Rv3651 | Rv3651 | 2.353359 |
| pknJ | Rv2088 | 2.34769 |
| Rv2874 | Rv2874 | 2.342568 |
| Rv2998 | Rv2998 | 2.341436 |
| Rv0040c | Rv0040c | 2.322871 |
| PPE | Rv3022c | 2.306015 |
| deoA | Rv3314c | 2.294118 |
| menB | Rv0548c | 2.293866 |
| mmaA2 | Rv0644c | 2.29151 |
| pheA | Rv3838c | 2.285353 |
| Rv1432 | Rv1432 | 2.276784 |
| Hypothetical protein | ORF08435 | 2.276168 |
| Rv03183 | Rv0318c | 2.274717 |
| Rv1191 | Rv1191 | 2.268336 |
| ketoacyl-CoA thiolase-related protein | ORF08252 | 2.263174 |
| Rv1971 | Rv1971 | 2.261841 |
| Rv3776 | Rv3776 | 2.257762 |
| Rv0592 | Rv0592 | 2.255669 |
| PE | Rv0335c | 2.254553 |
| Rv0744c | Rv0744c | 2.243251 |
| ilvB2 | Rv3470c | 2.232419 |
| Rv1889c | Rv1889c | 2.231127 |
| Rv3330 | Rv3330 | 2.229 |
| Rv0330c | Rv0330c | 2.227578 |
| plcA | Rv2351c | 2.211412 |
| PPE | Rv1790 | 2.204434 |
| Rv2370c | Rv2370c | 2.197039 |
| hypothetical protein | ORF05608 | 2.193718 |
| mercuric reductase/transcriptional regulator, fusion | ORF05528 | 2.190845 |
| Alr | Rv3423c | 2.183603 |
| Rv0494 | Rv0494 | 2.180863 |
| PE_PGRS | Rv0746 | 2.177101 |
| PE_PGRS | Rv0747 | 2.172893 |
| PE_PGRS | Rv0278c | 2.163296 |
| tlyA | Rv1694 | 2.159331 |
| Rv2013 | Rv2013 | 2.156244 |
| fadE13 | Rv0975c | 2.154061 |
| Rv2628 | Rv2628 | 2.141312 |
| Rv1888c | Rv1888c | 2.139152 |
| pgmA | Rv3068c | 2.126482 |
| Rv3548c | Rv3548c | 2.120194 |
| Rv3638 | Rv3638 | 2.119657 |
| moaB | Rv3110 | 2.118418 |
| rocE | Rv2320c | 2.116144 |
| Rv0395 | Rv0395 | 2.114499 |
| Rv0348 | Rv0348 | 2.108378 |
| Rv3768 | Rv3768 | 2.108052 |
| Rv0137c | Rv0137c | 2.096601 |
| hypothetical protein | ORFD0353 | 2.091969 |
| Rv0728c | Rv0728c | 2.09075 |
| Rv1672c | Rv1672c | 2.084831 |
| Rv2034 | Rv2034 | 2.077846 |
| Rv0023 | Rv0023 | 2.064448 |
| Rv3654c | Rv3654c | 2.057942 |
| Fpg | Rv2924c | 2.049801 |
| Rv0963c | Rv0963c | 2.046634 |
| Rv2016 | Rv2016 | 2.046164 |
| PPE family protein | ORF00805 | 2.040677 |
| Rv2560 | Rv2560 | 2.035737 |
| accD3 | Rv0904c | 2.031258 |
| Rv1422 | Rv1422 | 2.030543 |
| lppK | Rv2116 | 2.028884 |
| Rv2827c | Rv2827c | 2.027959 |
| Rv1742 | Rv1742 | 2.023653 |
| lipW | Rv0217c | 2.021559 |
| hypothetical protein | ORF03807 | 2.017235 |
| PE | Rv3622c | 2.01462 |
| Rv3386 | Rv3386 | 2.013194 |
| PPE | Rv1801 | 2.01106 |
| aceAa | Rv1915 | 2.003984 |
| fadE30 | Rv3560c | 2.001147 |
| hypothetical protein | ORF05916 | 2.000324 |
| Rv2489c | Rv2489c | 1.997982 |
| ureC | Rv1850 | 1.995461 |
| Rv2542 | Rv2542 | 1.992559 |
| Rv2507 | Rv2507 | 1.991956 |
| PPE | Rv0453 | 1.991513 |
| Rv1460 | Rv1460 | 1.987575 |
| panC | Rv3602c | 1.985515 |
| Rv2133c | Rv2133c | 1.985335 |
| aldA | Rv0768 | 1.983569 |
| Rv3862c | Rv3862c | 1.979609 |
| Rv1032c | Rv1032c | 1.97879 |
| Rv1137c | Rv1137c | 1.978733 |
| Rv1291c | Rv1291c | 1.960051 |
| rmlA | Rv0334 | 1.958063 |
| galK | Rv0620 | 1.956218 |
| Rv3912 | Rv3912 | 1.956043 |
| recC | Rv0631c | 1.955619 |
| Rv3575c | Rv3575c | 1.955139 |
| Rv0380c | Rv0380c | 1.944516 |
| Rv2657c | Rv2657c | 1.942944 |
| Rv2569c | Rv2569c | 1.937366 |
| hypothetical protein | ORF01318 | 1.936487 |
| Rv2674 | Rv2674 | 1.932519 |
| Rv1532c | Rv1532c | 1.929012 |
| Rv2974c | Rv2974c | 1.927332 |
| fadD1 | Rv1750c | 1.92309 |
| moxR2 | Rv3692 | 1.917227 |
| Rv0230c | Rv0230c | 1.913793 |
| Rv3090 | Rv3090 | 1.908482 |
| Rv2803c | Rv2803c | 1.905378 |
| Rv3121 | Rv3121 | 1.897796 |
| gabP | Rv0522 | 1.896125 |
| Rv2294 | Rv2294 | 1.895955 |
| nirB | Rv0252 | 1.888523 |
| Rv3727 | Rv3727 | 1.884468 |
| Rv1781c | Rv1781c | 1.880113 |
| hypothetical protein | ORFD0308 | 1.880052 |
| PE_PGRS | Rv0977 | 1.878651 |
| Rv1748 | Rv1748 | 1.877264 |
| Rv0674 | Rv0674 | 1.875861 |
| purC | Rv0780 | 1.874809 |
| Rv1945 | Rv1945 | 1.869857 |
| Rv0224c | Rv0224c | 1.861469 |
| mutA | Rv1492 | 1.857061 |
| Rv1395 | Rv1395 | 1.853699 |
| Rv2276 | Rv2276 | 1.852688 |
| Rv1004c | Rv1004c | 1.844548 |
| Rv3718c | Rv3718c | 1.841292 |
| Rv1414 | Rv1414 | 1.8404 |
| ctpA | Rv0092 | 1.840349 |
| Rv0310c | Rv0310c | 1.83947 |
| PPE | Rv0388c | 1.835715 |
| echA11 | Rv1141c | 1.831867 |
| Rv1003 | Rv1003 | 1.831613 |
| Rv1578c | Rv1578c | 1.819813 |
| Rv3422c | Rv3422c | 1.818123 |
| Rv1648 | Rv1648 | 1.815059 |
| transcriptional activator, LysR family, degenerate | ORF01880 | 1.812796 |
| Rv3669 | Rv3669 | 1.812097 |
| aldB | Rv3293 | 1.804906 |
| Rv0024 | Rv0024 | 1.79874 |
| Rv3729 | Rv3729 | 1.797528 |
| Rv2997 | Rv2997 | 1.795251 |
| ligB | Rv3062 | 1.795079 |
| Rv1965 | Rv1965 | 1.793793 |
| Rv2800 | Rv2800 | 1.792005 |
| whiB3 | Rv3416 | 1.780366 |
| Rv1242 | Rv1242 | 1.777011 |
| Rv0607 | Rv0607 | 1.774792 |
| Rv2170 | Rv2170 | 1.77399 |
| Rv2319c | Rv2319c | 1.757686 |
| Rv0193c | Rv0193c | 1.75596 |
| Rv1730c | Rv1730c | 1.755841 |
| Rv3643 | Rv3643 | 1.754082 |
| Rv3369 | Rv3369 | 1.752027 |
| uspC | Rv2318 | 1.749568 |
| Rv0264c | Rv0264c | 1.745941 |
| Rv1382 | Rv1382 | 1.745899 |
| Rv3835 | Rv3835 | 1.742663 |
| Rv1944c | Rv1944c | 1.737535 |
| Rv1580c | Rv1580c | 1.737223 |
| Rv2659c | Rv2659c | 1.735046 |
| cysS2 | Rv2130c | 1.732435 |
| Rv2510c | Rv2510c | 1.729131 |
| Rv0976c | Rv0976c | 1.72766 |
| Rv3261 | Rv3261 | 1.727013 |
| PPE | Rv0305c | 1.724857 |
| Rv2175c | Rv2175c | 1.721313 |
| Rv2100 | Rv2100 | 1.715136 |
| Rv3675 | Rv3675 | 1.711811 |
| Rv0599c | Rv0599c | 1.702195 |
| Rv1413 | Rv1413 | 1.700207 |
| Rv2577 | Rv2577 | 1.698678 |
| Rv0890c | Rv0890c | 1.697956 |
| Rv2224c | Rv2224c | 1.696872 |
| Rv0197 | Rv0197 | 1.696096 |
| Rv1455 | Rv1455 | 1.693064 |
| Rv0258c | Rv0258c | 1.690292 |
| grcC2 | Rv0989c | 1.68858 |
| rmlD | Rv3266c | 1.680405 |
| Rv0228 | Rv0228 | 1.679758 |
| pknL | Rv2176 | 1.678872 |
| PE_PGRS | Rv2615c | 1.673569 |
| Rv0739 | Rv0739 | 1.662016 |
| embR | Rv1267c | 1.65734 |
| hypothetical protein | ORFD0106 | 1.65702 |
| PE_PGRS family protein | ORF03593 | 1.656412 |
| end | Rv0670 | 1.65476 |
| Rv0227c | Rv0227c | 1.654604 |
| PPE | Rv2123 | 1.653573 |
| Rv0398c | Rv0398c | 1.652581 |
| Rv0621 | Rv0621 | 1.652116 |
| lipB | Rv2217 | 1.650841 |
| fabD2 | Rv0649 | 1.645011 |
| Rv3088 | Rv3088 | 1.640762 |
| Rv3639c | Rv3639c | 1.635291 |
| Rv3807c | Rv3807c | 1.634857 |
| moaC3 | Rv3324c | 1.63079 |
| Rv1218c | Rv1218c | 1.630368 |
| mpt70 | Rv2875 | 1.629237 |
| nrdZ | Rv0570 | 1.62829 |
| Rv3745c | Rv3745c | 1.621602 |
| Rv3067 | Rv3067 | 1.618251 |
| Rv1804c | Rv1804c | 1.618223 |
| Rv0887c | Rv0887c | 1.610527 |
| smc | Rv2922c | 1.608194 |
| Rv0116c | Rv0116c | 1.606608 |
| Rv1084 | Rv1084 | 1.602394 |
| Rv0881 | Rv0881 | 1.598841 |
| Rv3831 | Rv3831 | 1.592794 |
| hypothetical protein | ORFD0023 | 1.590342 |
| Rv0544c | Rv0544c | 1.590086 |
| Rv3399 | Rv3399 | 1.588863 |
| Rv1982c | Rv1982c | 1.584112 |
| Rv2514c | Rv2514c | 1.581057 |
| Rv0738 | Rv0738 | 1.577886 |
| hypothetical protein | ORF01236 | 1.576972 |
| Rv0914c | Rv0914c | 1.574293 |
| ppsC | Rv2933 | 1.573593 |
| murE | Rv2158c | 1.571917 |
| Rv3792 | Rv3792 | 1.56777 |
| Rv2563 | Rv2563 | 1.56483 |
| Rv3760 | Rv3760 | 1.556001 |
| hypothetical protein | ORF05893 | 1.552785 |
| ephC | Rv1124 | 1.550388 |
| Rv1739c | Rv1739c | 1.546327 |
| Rv2717c | Rv2717c | 1.522659 |
| Rv3679 | Rv3679 | 1.518195 |
| Rv3553 | Rv3553 | 1.515371 |
| Rv1634 | Rv1634 | 1.512092 |
| hycD | Rv0084 | 1.511735 |
| ligC | Rv3731 | 1.511174 |
| Rv0375c | Rv0375c | 1.505545 |
| hypothetical protein | ORF01611 | 1.497301 |
| Rv3496c | Rv3496c | 1.495862 |
| Rv2545 | Rv2545 | 1.490187 |
| aspB | Rv3565 | 1.480652 |
| Rv3445c | Rv3445c | 1.47267 |
| trmD | Rv2906c | 1.467415 |
| Rv3359 | Rv3359 | 1.463844 |
| PE_PGRS | Rv1441c | 1.448083 |
| Rv0521c | Rv0521c | 1.445041 |
| Rv0927c | Rv0927c | 1.421682 |
| Rv3741c | Rv3741c | 1.418835 |
| add | Rv3313c | 1.412321 |
| lppY | Rv2999 | 1.410681 |
| Rv2041c | Rv2041c | 1.388988 |
| embA | Rv3794 | 1.342046 |
| Rv0303 | Rv0303 | 1.336156 |
| Rv1767 | Rv1767 | 1.318372 |
