# Supplementary material for: Transcriptome analysis of mycobacteria in sputum samples of pulmonary tuberculosis patients
Source: PLoS One. 2017 Mar 10;12(3):e0173508. doi: 10.1371/journal.pone.0173508 (PMC5345810; doi:10.1371/journal.pone.0173508)
Supplement: S3 Table — (DOCX) [file pone.0173508.s003.docx]

**S3 Table: Downregulated mycobacterial genes in sputum of smear positive patients**

| **Name** | **ID** | **Fold Change** |
| --- | --- | --- |
| hypothetical protein | ORFD0123 | -102.39 |
| efpA | Rv2846c | -79.1086 |
| hypothetical protein | ORFD0122 | -19.1165 |
| Rv3620c | Rv3620c | -18.315 |
| hypothetical protein | ORFD0214 | -16.1261 |
| rpmE | Rv1298 | -16.0523 |
| Rv3582c | Rv3582c | -14.0402 |
| hypothetical protein | ORF04157 | -13.6811 |
| Rv1828 | Rv1828 | -12.8742 |
| rpsJ | Rv0700 | -12.8256 |
| bfrB | Rv3841 | -11.7965 |
| Rv3686c | Rv3686c | -11.4383 |
| Rv2204c | Rv2204c | -11.3203 |
| Rv1792 | Rv1792 | -10.435 |
| hupB | Rv2986c | -10.3461 |
| desA2 | Rv1094 | -10.1088 |
| PPE | Rv3478 | -9.54369 |
| acpM | Rv2244 | -9.41436 |
| Rv1793 | Rv1793 | -9.30495 |
| Rv1871c | Rv1871c | -9.2518 |
| nuoH | Rv3152 | -9.19952 |
| Rv3616c | Rv3616c | -9.18324 |
| ribH | Rv1416 | -9.16836 |
| qcrA | Rv2195 | -9.07643 |
| Rv1830 | Rv1830 | -9.07322 |
| Rv1398c | Rv1398c | -8.7745 |
| atpD | Rv1310 | -8.72551 |
| nuoM | Rv3157 | -8.70504 |
| esat6 | Rv3875 | -8.31785 |
| Rv3922c | Rv3922c | -8.26616 |
| desA3 | Rv3229c | -8.26605 |
| rpmD | Rv0722 | -8.15474 |
| nuoN | Rv3158 | -8.13031 |
| Rv2751 | Rv2751 | -8.05079 |
| Rv3798 | Rv3798 | -7.95973 |
| Rv0336 | Rv0336 | -7.73406 |
| PE_PGRS | Rv2396 | -7.69382 |
| Rv1827 | Rv1827 | -7.65703 |
| rnpA | Rv3923c | -7.37054 |
| rpsL | Rv0682 | -7.30891 |
| Rv2438c | Rv2438c | -6.5909 |
| hypothetical protein | ORF03834 | -6.48147 |
| Rv3281 | Rv3281 | -6.42466 |
| rplA | Rv0641 | -6.32242 |
| Rv2347c | Rv2347c | -6.22228 |
| Rv1197 | Rv1197 | -6.21148 |
| Rv1884c | Rv1884c | -5.93761 |
| Rv3408 | Rv3408 | -5.91656 |
| ctaE | Rv2193 | -5.87287 |
| nrdE | Rv3051c | -5.8184 |
| lgt | Rv1614 | -5.78146 |
| Rv0170 | Rv0170 | -5.70331 |
| Rv1904 | Rv1904 | -5.64226 |
| PE | Rv1386 | -5.61824 |
| Rv1397c | Rv1397c | -5.57081 |
| trpC | Rv1611 | -5.55052 |
| Rv2190c | Rv2190c | -5.53366 |
| fbpA | Rv3804c | -5.52614 |
| nirA | Rv2391 | -5.5205 |
| Rv0298 | Rv0298 | -5.52036 |
| rpmA | Rv2441c | -5.51322 |
| Rv2137c | Rv2137c | -5.49909 |
| cspA | Rv3648c | -5.37675 |
| fadD28 | Rv2941 | -5.36715 |
| Rv0038 | Rv0038 | -5.35256 |
| rplC | Rv0701 | -5.33056 |
| Rv3282 | Rv3282 | -5.26068 |
| Rv2949c | Rv2949c | -5.25501 |
| Rv2199c | Rv2199c | -5.25145 |
| qcrB | Rv2196 | -5.23916 |
| lipF | Rv3487c | -5.15956 |
| conserved hypothetical protein | ORF01755 | -5.13183 |
| Rv2128 | Rv2128 | -5.1108 |
| kasB | Rv2246 | -5.09205 |
| Rv1038c | Rv1038c | -5.08038 |
| drrB | Rv2937 | -5.034 |
| rplQ | Rv3456c | -4.96765 |
| lppW | Rv2905 | -4.96448 |
| nusG | Rv0639 | -4.94714 |
| Rv0288 | Rv0288 | -4.85512 |
| Rv1747 | Rv1747 | -4.84835 |
| Rv2474c | Rv2474c | -4.82769 |
| sigB | Rv2710 | -4.81784 |
| rpmJ | Rv3461c | -4.80792 |
| rne | Rv2444c | -4.80097 |
| atpB | Rv1304 | -4.794 |
| desA1 | Rv0824c | -4.75917 |
| Rv2602 | Rv2602 | -4.68899 |
| infA | Rv3462c | -4.66961 |
| Rv1698 | Rv1698 | -4.65955 |
| menG | Rv3853 | -4.59014 |
| Rv2367c | Rv2367c | -4.59014 |
| Rv2376c | Rv2376c | -4.58267 |
| Rv3583c | Rv3583c | -4.58209 |
| Rv0174 | Rv0174 | -4.57483 |
| greA | Rv1080c | -4.56062 |
| Rv0248c | Rv0248c | -4.55239 |
| nrdG | Rv3048c | -4.49362 |
| cbhK | Rv2202c | -4.49329 |
| Rv3220c | Rv3220c | -4.46244 |
| groES | Rv3418c | -4.44251 |
| nadC | Rv1596 | -4.41313 |
| cysH | Rv2392 | -4.40983 |
| Rv0636 | Rv0636 | -4.37954 |
| PE | Rv1195 | -4.35374 |
| tsnR | Rv1644 | -4.33047 |
| Rv1856c | Rv1856c | -4.2916 |
| aroG | Rv2178c | -4.26891 |
| Rv1219c | Rv1219c | -4.25694 |
| umaA1 | Rv0469 | -4.22359 |
| hypothetical protein | ORF01824 | -4.21331 |
| Rv0655 | Rv0655 | -4.18312 |
| dnaE1 | Rv1547 | -4.17452 |
| rplO | Rv0723 | -4.16272 |
| rplB | Rv0704 | -4.1575 |
| rplE | Rv0716 | -4.1356 |
| rplD | Rv0702 | -4.12505 |
| atpG | Rv1309 | -4.12125 |
| ahpE | Rv2238c | -4.10897 |
| choD | Rv3409c | -4.10816 |
| Rv3479 | Rv3479 | -4.10607 |
| Rv3412 | Rv3412 | -4.0927 |
| clpX | Rv2457c | -4.08219 |
| Rv1313c | Rv1313c | -4.08021 |
| Rv0659c | Rv0659c | -4.06335 |
| Rv3778c | Rv3778c | -4.04711 |
| Rv3874 | Rv3874 | -4.04598 |
| fadD22 | Rv2948c | -4.04397 |
| fadD31 | Rv1925 | -4.01338 |
| rpoC | Rv0668 | -4.01019 |
| ansP | Rv2127 | -4.00543 |
| dsbF | Rv1677 | -3.97917 |
| Rv2553c | Rv2553c | -3.9459 |
| rho | Rv1297 | -3.93237 |
| phoP | Rv0757 | -3.91978 |
| nrdH | Rv3053c | -3.84151 |
| hypothetical protein | ORF06113 | -3.80698 |
| echA3 | Rv0632c | -3.7969 |
| trpA | Rv1613 | -3.78277 |
| Rv3920c | Rv3920c | -3.77389 |
| Rv3252c | Rv3252c | -3.76441 |
| Rv3613c | Rv3613c | -3.75779 |
| gid | Rv3919c | -3.7263 |
| drrA | Rv2936 | -3.70909 |
| Rv1887 | Rv1887 | -3.69901 |
| Rv1615 | Rv1615 | -3.68544 |
| fadD26 | Rv2930 | -3.65737 |
| PE | Rv3872 | -3.6532 |
| Rv0637 | Rv0637 | -3.63612 |
| Rv1037c | Rv1037c | -3.62441 |
| inhA | Rv1484 | -3.6208 |
| lldD1 | Rv0694 | -3.62012 |
| Rv2516c | Rv2516c | -3.61947 |
| rpsS | Rv0705 | -3.61231 |
| atpH | Rv1307 | -3.60565 |
| ponA | Rv0050 | -3.59966 |
| Rv0462 | Rv0462 | -3.57502 |
| Rv1312 | Rv1312 | -3.55717 |
| Rv0284 | Rv0284 | -3.53876 |
| Rv3783 | Rv3783 | -3.53338 |
| Rv3854c | Rv3854c | -3.50823 |
| PE | Rv3477 | -3.50245 |
| hypothetical protein | ORFD0024 | -3.49448 |
| conserved hypothetical protein | ORF03148 | -3.4902 |
| Rv3878 | Rv3878 | -3.48577 |
| fabG4 | Rv0242c | -3.48012 |
| Rv2115c | Rv2115c | -3.46459 |
| fusA | Rv0684 | -3.46214 |
| clpC | Rv3596c | -3.45424 |
| fadB4 | Rv3141 | -3.44812 |
| Rv1794 | Rv1794 | -3.40587 |
| conserved hypothetical protein | ORF01756 | -3.40556 |
| Rv3130c | Rv3130c | -3.40509 |
| Rv1222 | Rv1222 | -3.40345 |
| whiB1 | Rv3219 | -3.39165 |
| secF | Rv2586c | -3.38553 |
| pstA2 | Rv0936 | -3.38262 |
| adhA | Rv1862 | -3.37646 |
| Rv2203 | Rv2203 | -3.37576 |
| nuoK | Rv3155 | -3.36016 |
| Rv1086 | Rv1086 | -3.35556 |
| Rv0569 | Rv0569 | -3.34704 |
| Rv3808c | Rv3808c | -3.34299 |
| Rv2603c | Rv2603c | -3.33584 |
| Rv0692 | Rv0692 | -3.33249 |
| rpoB | Rv0667 | -3.32862 |
| hspX | Rv2031c | -3.31035 |
| pstS | Rv0932c | -3.30388 |
| nrdI | Rv3052c | -3.29716 |
| Rv0178 | Rv0178 | -3.29152 |
| Rv3049c | Rv3049c | -3.27496 |
| Rv1772 | Rv1772 | -3.25903 |
| lppU | Rv2784c | -3.24521 |
| Rv1502 | Rv1502 | -3.24159 |
| moaC2 | Rv0864 | -3.2326 |
| gcpE | Rv2868c | -3.2306 |
| Rv0190 | Rv0190 | -3.22519 |
| Rv3142c | Rv3142c | -3.21257 |
| Rv3619c | Rv3619c | -3.20162 |
| Rv1810 | Rv1810 | -3.1952 |
| hypothetical protein | ORF04372 | -3.17008 |
| phoH2 | Rv1095 | -3.16336 |
| ndkA | Rv2445c | -3.15544 |
| Rv3755c | Rv3755c | -3.15282 |
| Rv3592 | Rv3592 | -3.14757 |
| ppsE | Rv2935 | -3.14103 |
| Rv2840c | Rv2840c | -3.12744 |
| atpA | Rv1308 | -3.10283 |
| Rv2779c | Rv2779c | -3.10246 |
| sigA | Rv2703 | -3.09877 |
| pknD | Rv0931c | -3.09874 |
| Rv1461 | Rv1461 | -3.09573 |
| PPE | Rv0286 | -3.09294 |
| hypothetical protein | ORFD0232 | -3.08833 |
| pvdS | Rv3232c | -3.04846 |
| Rv3295 | Rv3295 | -3.04214 |
| Rv1478 | Rv1478 | -3.03891 |
| Rv2297 | Rv2297 | -3.03842 |
| pstA1 | Rv0930 | -3.03495 |
| Rv2959c | Rv2959c | -2.99863 |
| secG | Rv1440 | -2.99491 |
| rpmH | Rv3924c | -2.9888 |
| PPE | Rv3429 | -2.9753 |
| Rv3212 | Rv3212 | -2.96672 |
| Rv0060 | Rv0060 | -2.96385 |
| rplK | Rv0640 | -2.96381 |
| rodA | Rv0017c | -2.95593 |
| mpt64 | Rv1980c | -2.95429 |
| rpsQ | Rv0710 | -2.94977 |
| Rv3662c | Rv3662c | -2.94749 |
| Rv2968c | Rv2968c | -2.93393 |
| Hypothetical protein | ORF08412 | -2.90722 |
| Rv0741 | Rv0741 | -2.90678 |
| Rv0426c | Rv0426c | -2.9058 |
| Rv3687c | Rv3687c | -2.8929 |
| atpF | Rv1306 | -2.89092 |
| parA | Rv3917c | -2.88797 |
| Rv1339 | Rv1339 | -2.88514 |
| Rv1795 | Rv1795 | -2.88272 |
| Rv3678c | Rv3678c | -2.86636 |
| Rv1639c | Rv1639c | -2.86259 |
| Rv1869c | Rv1869c | -2.85134 |
| obg | Rv2440c | -2.84733 |
| Rv0430 | Rv0430 | -2.84342 |
| lytB' | Rv1110 | -2.83139 |
| rplI | Rv0056 | -2.83085 |
| Rv0383c | Rv0383c | -2.82192 |
| Rv2792c | Rv2792c | -2.80291 |
| secD | Rv2587c | -2.78933 |
| Rv1697 | Rv1697 | -2.78242 |
| ilvC | Rv3001c | -2.77634 |
| serB2 | Rv3042c | -2.77472 |
| Rv3680 | Rv3680 | -2.7681 |
| ogt | Rv1316c | -2.7581 |
| Rv1783 | Rv1783 | -2.7568 |
| ephE | Rv3670 | -2.749 |
| Rv0314c | Rv0314c | -2.74789 |
| Rv3127 | Rv3127 | -2.74615 |
| Rv2111c | Rv2111c | -2.73791 |
| Rv0301 | Rv0301 | -2.70876 |
| atpE | Rv1305 | -2.70676 |
| Rv0292 | Rv0292 | -2.70573 |
| fadD29 | Rv2950c | -2.70322 |
| Rv1813c | Rv1813c | -2.6894 |
| Rv0543c | Rv0543c | -2.68033 |
| Rv1054 | Rv1054 | -2.67865 |
| htrA | Rv1223 | -2.67521 |
| Rv2752c | Rv2752c | -2.66795 |
| Rv1324 | Rv1324 | -2.6667 |
| Rv0010c | Rv0010c | -2.66229 |
| clpP | Rv2461c | -2.64979 |
| rplP | Rv0708 | -2.64844 |
| Rv2010 | Rv2010 | -2.64451 |
| cdsA | Rv2881c | -2.61632 |
| Rv3143 | Rv3143 | -2.60742 |
| kasA | Rv2245 | -2.60709 |
| Rv2094c | Rv2094c | -2.60377 |
| Rv1898 | Rv1898 | -2.6029 |
| Rv2258c | Rv2258c | -2.60273 |
| Rv0605 | Rv0605 | -2.58247 |
| Rv2395 | Rv2395 | -2.58198 |
| fadB | Rv0860 | -2.58194 |
| Rv3849 | Rv3849 | -2.57831 |
| excisionase, putative | ORF05040 | -2.56159 |
| Rv2102 | Rv2102 | -2.56071 |
| Rv1073 | Rv1073 | -2.55328 |
| Rv1883c | Rv1883c | -2.54105 |
| Rv1390 | Rv1390 | -2.52989 |
| Rv2707 | Rv2707 | -2.52778 |
| Rv1050 | Rv1050 | -2.50924 |
| Rv0073 | Rv0073 | -2.49722 |
| Rv1751 | Rv1751 | -2.49557 |
| pks12 | Rv2048c | -2.49375 |
| metZ | Rv0391 | -2.48393 |
| Rv0870c | Rv0870c | -2.47359 |
| Rv3909 | Rv3909 | -2.47052 |
| Rv0282 | Rv0282 | -2.4663 |
| narH | Rv1162 | -2.46411 |
| nuoG | Rv3151 | -2.46062 |
| rpsK | Rv3459c | -2.45964 |
| ephD | Rv2214c | -2.45906 |
| rpsG | Rv0683 | -2.45003 |
| Hypothetical protein | ORF08416 | -2.44896 |
| lepB | Rv2903c | -2.4349 |
| pknH | Rv1266c | -2.43058 |
| Rv2393 | Rv2393 | -2.42702 |
| mmpL8 | Rv3823c | -2.42646 |
| dapB | Rv2773c | -2.42421 |
| cmk | Rv1712 | -2.41526 |
| Rv1156 | Rv1156 | -2.40586 |
| Rv0466 | Rv0466 | -2.40521 |
| galE1 | Rv0501 | -2.40459 |
| Rv1487 | Rv1487 | -2.40394 |
| fadD2 | Rv0270 | -2.40183 |
| gyrA | Rv0006 | -2.39135 |
| PPE | Rv1387 | -2.3888 |
| rpsI | Rv3442c | -2.37343 |
| ppp | Rv0018c | -2.35991 |
| Rv0229c | Rv0229c | -2.35903 |
| Rv3024c | Rv3024c | -2.35605 |
| Rv2136c | Rv2136c | -2.35525 |
| wag31 | Rv2145c | -2.35499 |
| Rv3581c | Rv3581c | -2.35464 |
| Rv1616 | Rv1616 | -2.35048 |
| Rv1149 | Rv1149 | -2.33103 |
| purQ | Rv0788 | -2.31953 |
| pterin-4-alpha-carbinolamine dehydratase, putative | ORF06120 | -2.29582 |
| Rv1322 | Rv1322 | -2.28133 |
| pncA | Rv2043c | -2.27938 |
| Rv2302 | Rv2302 | -2.27825 |
| prfA | Rv1299 | -2.27796 |
| Rv0364 | Rv0364 | -2.27169 |
| Rv1929c | Rv1929c | -2.2672 |
| metB | Rv1079 | -2.26695 |
| Rv2255c | Rv2255c | -2.25884 |
| Rv2898c | Rv2898c | -2.25567 |
| Rv3771c | Rv3771c | -2.24238 |
| Rv2402 | Rv2402 | -2.24036 |
| efp | Rv2534c | -2.23755 |
| Rv0474 | Rv0474 | -2.2372 |
| Rv3278c | Rv3278c | -2.22176 |
| Rv2729c | Rv2729c | -2.21383 |
| PPE | Rv2430c | -2.19314 |
| fdhD | Rv2899c | -2.19027 |
| bglS | Rv0186 | -2.1821 |
| Rv1625c | Rv1625c | -2.17556 |
| Rv0299 | Rv0299 | -2.17479 |
| phoY2 | Rv0821c | -2.17254 |
| Rv1102c | Rv1102c | -2.17194 |
| fadE22 | Rv3061c | -2.16691 |
| Rv1009 | Rv1009 | -2.15288 |
| Rv3263 | Rv3263 | -2.14943 |
| Rv1597 | Rv1597 | -2.14561 |
| Rv1464 | Rv1464 | -2.14005 |
| Rv0276 | Rv0276 | -2.13386 |
| Rv3871 | Rv3871 | -2.12789 |
| moeZ | Rv3206c | -2.12711 |
| Rv0080 | Rv0080 | -2.11975 |
| mmpS5 | Rv0677c | -2.11974 |
| whiB2 | Rv3260c | -2.11736 |
| mmpL7 | Rv2942 | -2.11593 |
| rplF | Rv0719 | -2.11111 |
| Rv0587 | Rv0587 | -2.10992 |
| fadE25 | Rv3274c | -2.10809 |
| Rv1841c | Rv1841c | -2.10505 |
| Rv0114 | Rv0114 | -2.09942 |
| Rv1534 | Rv1534 | -2.09581 |
| Rv1233c | Rv1233c | -2.09443 |
| pfkA | Rv3010c | -2.09117 |
| tig | Rv2462c | -2.09004 |
| Rv0472c | Rv0472c | -2.07625 |
| lpqF | Rv3593 | -2.0722 |
| accD4 | Rv3799c | -2.07152 |
| glnA2 | Rv2222c | -2.06815 |
| Rv1404 | Rv1404 | -2.06167 |
| plsB2 | Rv2482c | -2.05954 |
| Rv1097c | Rv1097c | -2.05857 |
| Rv0556 | Rv0556 | -2.0582 |
| Rv3677c | Rv3677c | -2.05203 |
| Rv0140 | Rv0140 | -2.04579 |
| Rv2189c | Rv2189c | -2.04349 |
| purM | Rv0809 | -2.0415 |
| Rv0658c | Rv0658c | -2.03975 |
| grpE | Rv0351 | -2.03324 |
| lipN | Rv2970c | -2.02462 |
| Rv0205 | Rv0205 | -2.0219 |
| Rv2166c | Rv2166c | -2.02043 |
| hisC | Rv1600 | -2.0202 |
| Rv1780 | Rv1780 | -2.02002 |
| Rv2140c | Rv2140c | -2.01785 |
| hypothetical protein | ORF04174 | -2.01097 |
| adhC | Rv3045 | -2.00924 |
| glpK | Rv3696c | -2.00741 |
| Rv3781 | Rv3781 | -2.00362 |
| tagA | Rv1210 | -1.99599 |
| Rv2818c | Rv2818c | -1.98955 |
| Rv2954c | Rv2954c | -1.97861 |
| PPE | Rv1789 | -1.97773 |
| echA12 | Rv1472 | -1.96272 |
| Rv3269 | Rv3269 | -1.96138 |
| eno | Rv1023 | -1.9609 |
| fadE20 | Rv2724c | -1.95975 |
| amiB | Rv3306c | -1.95905 |
| Rv2254c | Rv2254c | -1.95674 |
| Rv2743c | Rv2743c | -1.95415 |
| gltA2 | Rv0896 | -1.95331 |
| Rv0239 | Rv0239 | -1.93866 |
| dfp | Rv1391 | -1.93802 |
| Hypothetical protein | ORF08400 | -1.92925 |
| Rv2181 | Rv2181 | -1.92916 |
| Rv1053c | Rv1053c | -1.91622 |
| Rv2745c | Rv2745c | -1.90797 |
| rfbE | Rv3782 | -1.90163 |
| hypothetical protein | ORFD0182 | -1.89983 |
| Rv2990c | Rv2990c | -1.89232 |
| hypothetical protein | ORF01330 | -1.88534 |
| dxs | Rv2682c | -1.87214 |
| Rv0588 | Rv0588 | -1.8595 |
| Rv0912 | Rv0912 | -1.8555 |
| hisF | Rv1605 | -1.85052 |
| narI | Rv1164 | -1.85023 |
| rpmC | Rv0709 | -1.8433 |
| Rv1919c | Rv1919c | -1.83486 |
| Rv3723 | Rv3723 | -1.82931 |
| Rv2675c | Rv2675c | -1.82024 |
| Rv3767c | Rv3767c | -1.81323 |
| Rv2944 | Rv2944 | -1.80712 |
| Rv0431 | Rv0431 | -1.80208 |
| pitA | Rv0545c | -1.80161 |
| Rv1885c | Rv1885c | -1.79845 |
| Rv2082 | Rv2082 | -1.78989 |
| Rv2536 | Rv2536 | -1.77343 |
| gpsI | Rv2783c | -1.766 |
| drrC | Rv2938 | -1.75901 |
| Rv3791 | Rv3791 | -1.75863 |
| Rv3766 | Rv3766 | -1.74854 |
| pykA | Rv1617 | -1.72835 |
| Rv3574 | Rv3574 | -1.72772 |
| Rv0287 | Rv0287 | -1.72109 |
| Rv1019 | Rv1019 | -1.71117 |
| Rv0241c | Rv0241c | -1.70919 |
| Rv1488 | Rv1488 | -1.69824 |
| Rv1891 | Rv1891 | -1.69558 |
| Rv1144 | Rv1144 | -1.6777 |
| fadA3 | Rv1074c | -1.67573 |
| PE_PGRS | Rv0578c | -1.67219 |
| accD6 | Rv2247 | -1.67199 |
| Rv1545 | Rv1545 | -1.66601 |
| fbpB | Rv1886c | -1.66464 |
| arsB | Rv2685 | -1.65537 |
| Rv2530c | Rv2530c | -1.64429 |
| Rv2129c | Rv2129c | -1.64238 |
| Rv1011 | Rv1011 | -1.62701 |
| hypothetical protein | ORFD0292 | -1.59774 |
| Rv1287 | Rv1287 | -1.5905 |
| pstC | Rv0935 | -1.58068 |
| Rv2390c | Rv2390c | -1.53858 |
| helZ | Rv2101 | -1.53385 |
| Rv3805c | Rv3805c | -1.53202 |
| Rv1043c | Rv1043c | -1.52037 |
| echA1 | Rv0222 | -1.50853 |
| Rv3407 | Rv3407 | -1.50352 |
| gcvH | Rv1826 | -1.47599 |
| Rv1008 | Rv1008 | -1.47342 |
| Rv2588c | Rv2588c | -1.45443 |
| hypothetical protein | ORF04486 | -1.42269 |
| Rv1024 | Rv1024 | -1.39353 |
| ccsA | Rv0527 | -1.37946 |
| cwlM | Rv3915 | -1.25611 |
