# Supplementary material for: Transcriptome analysis of mycobacteria in sputum samples of pulmonary tuberculosis patients
Source: PLoS One. 2017 Mar 10;12(3):e0173508. doi: 10.1371/journal.pone.0173508 (PMC5345810; doi:10.1371/journal.pone.0173508)
Supplement: S4 Table — (DOCX) [file pone.0173508.s004.docx]

| **Functional Group** | **Description/Association** | **# of Genes** | **Names of differentially expressed genes in functional category** |
| --- | --- | --- | --- |
| 30S ribosomal proteins (22)^a^ | Protein synthesis | 7(down) | Rv0683(rpsG), Rv3442c (rpsI), Rv0700 (rpsJ), Rv0682 (rpsL), Rv0705 (rpsS), Rv0710 (rpsQ), Rv3459c ( rpsK) |
| 50S ribosomal proteins (36) | Protein synthesis | 16 (down) | Rv2441c (rpmA), Rv0722 (rpmD), Rv1298 (rpmE), Rv3924c (rpmH), Rv3461c (rpmJ), Rv0641 (rplA), Rv0704 (rplB), Rv0701 (rplC), Rv0702 (rplD), Rv0716 (rplE), Rv0719 (rplF), Rv0056 (rplI), Rv0640 (rplK), Rv0723 (rplO), Rv0708 (rplP), Rv3456c (rplQ), |
| ATP Synthase (8) | Energy | 7 (down) | *Rv1308 (atpA),* *Rv1304 (atpB),* *Rv1310 (atpD),*  *Rv1305 (atpE),*  *Rv1306 (atpF),*  *Rv1309 (atpG),*  *Rv1307(atpH)* |

**S4 Table: Identity of differentially expressed functional category genes indicative of *M. tb* replication**

^a^ Number within parentheses indicates total number of genes in the *M. tb* genome within this functional group
