# Supplementary material for: Transcriptome analysis of mycobacteria in sputum samples of pulmonary tuberculosis patients
Source: PLoS One. 2017 Mar 10;12(3):e0173508. doi: 10.1371/journal.pone.0173508 (PMC5345810; doi:10.1371/journal.pone.0173508)
Supplement: S5 Table — (DOCX) [file pone.0173508.s005.docx]

**Table S5: Identity of differentially expressed functional category genes indicative of *M. tb* respiratory state**

| **Functional Group** | **Description/Association** | **# of Genes** | **Names of differentially expressed genes in functional category** |
| --- | --- | --- | --- |
| NADH DH1 (14)^a^ | Aerobic respiration | 5 (down) | *Rv3151(nuoG),*  *Rv3152 (nuoH),*  *Rv3157 (nuoM),*  *Rv3158 (nuoN),*  *Rv3155 (nuoK)* |
| Cytochrome c Reductase (3) | Aerobic respiration | 2 (down) | *Rv2195 (qcrA),*  *Rv2196 (qcrB)* |
| Cytochrome c Oxidase (4) | Aerobic respiration | 1 (down) | *Rv2193 (ctaE)* |
| NADH DH2 (2) | Alternative Electron Transfer | NDE^c^ |  |
| Nitrate Reductase & Transport (5) | Non-aerobic respiration | 1 (down) | *Rv1162 (narH)* |

^a^ Number within parentheses indicates total number of genes in the *M. tb* genome within this functional group

^c^not differentially expressed
