# Supplementary material for: Transcriptome analysis of mycobacteria in sputum samples of pulmonary tuberculosis patients
Source: PLoS One. 2017 Mar 10;12(3):e0173508. doi: 10.1371/journal.pone.0173508 (PMC5345810; doi:10.1371/journal.pone.0173508)
Supplement: S6 Table — (DOCX) [file pone.0173508.s006.docx]

**S6 Table: Identity of differentially expressed virulence-associated cell wall synthesis and transport genes**

| **Functional Group** | **Description/Association** | **# of Genes** | **Names of differentially expressed genes in functional category** |
| --- | --- | --- | --- |
| PDIM/PGL Synthesis/transport (36)^a^ | Cell wall/ virulence | 9 (down) | *Rv2930 (fadD26), Rv2935 (ppsE), Rv2936 (drrA), Rv2937 (drrB), Rv2941 (fadD28), Rv2942 (mmpL7), Rv2948c (fadD22), Rv2950c (fadD29), Rv2959c* |

^a^ Number within parentheses indicates total number of genes in the *M. tb* genome within this functional group
