# Supplementary material for: Transcriptome analysis of mycobacteria in sputum samples of pulmonary tuberculosis patients
Source: PLoS One. 2017 Mar 10;12(3):e0173508. doi: 10.1371/journal.pone.0173508 (PMC5345810; doi:10.1371/journal.pone.0173508)
Supplement: S7 Table — (DOCX) [file pone.0173508.s007.docx]

**S7 Table: Identity of differentially expressed genes of *M. tb* sec and tat secretory pathways**

| **Functional Group** | **Description/Association** | **# of Genes** | **Names of differentially expressed genes in functional category** |
| --- | --- | --- | --- |
| Sec pathway (8)^a^ | Protein secretion | 3 (down) | *Rv1440(secG), Rv2586c(secF), Rv2587c(* *secD)* |
| Tat pathway (4) | Protein secretion | 1 (down) | *Rv2094c* |

^a^ Number within parentheses indicates total number of genes in the *M. tb* genome within this functional group
