# Supplementary material for: Transcriptome analysis of mycobacteria in sputum samples of pulmonary tuberculosis patients
Source: PLoS One. 2017 Mar 10;12(3):e0173508. doi: 10.1371/journal.pone.0173508 (PMC5345810; doi:10.1371/journal.pone.0173508)
Supplement: S8 Table — (DOCX) [file pone.0173508.s008.docx]

**S8 Table: Identity of differentially expressed genes located within ESX type VII secretion system loci**

| **Functional Group** | **Description/Association** | **# of Genes** | **Names of differentially expressed genes in functional category** |
| --- | --- | --- | --- |
| ESX-1 (20)^a^ | ESAT-6 secretion | 5 (down) | *Rv3871, Rv3872, Rv3875(esat6), Rv3874 (cfp10), Rv3878* |
| ESX-2 (12) | Unknown | NDE |  |
| ESX-3 (11) | Iron/Zinc homeostasis | 5 (down) | *Rv0282, Rv0284, Rv0286, Rv0288, Rv0292* |
| ESX-4 (7) | Most ancestral | NDE |  |
| ESX-5 (17) | PE/PPE secretion | 4 (down) | *Rv1783, Rv1792 , Rv1793 , Rv1795* |

^a^ Number within parentheses indicates total number of genes in the *M. tb* genome within this functional group
