# Supplementary material for: Transcriptome analysis of mycobacteria in sputum samples of pulmonary tuberculosis patients
Source: PLoS One. 2017 Mar 10;12(3):e0173508. doi: 10.1371/journal.pone.0173508 (PMC5345810; doi:10.1371/journal.pone.0173508)
Supplement: S9 Table — (DOCX) [file pone.0173508.s009.docx]

**S9 Table: Identity of differentially expressed genes encoding ESAT-6-like proteins**

| **Functional Group** | **Description/Association** | **# of Genes** | **Names of differentially expressed genes in functional category** |
| --- | --- | --- | --- |
| Total ESAT-6-like in genome (23)^a^ | Similar structure to ESAT-6 | 11 (down) | *Rv3875 (esxA), Rv3874 (esxB), Rv0288c (esxH), Rv1037c (esxI), Rv1792 (esxM), Rv1793 (esxN), Rv1038c (esxJ), Rv1197 (esxK), Rv2347c (esxP), Rv3619c (esxV), Rv3620c (esxW)* |
| ESAT-6-like Outside ESX loci (13) | Do not reside in ESX loci | 6 (down) | *Rv1037c (esxI),Rv1038c (esxJ), Rv1197 (esxK), Rv2347c (esxP), Rv3619c (esxV), Rv3620c (esxW)* |

^a^ Number within parentheses indicates total number of genes in the *M. tb* genome within this functional group
