# Supplementary material for: Transcriptome analysis of mycobacteria in sputum samples of pulmonary tuberculosis patients
Source: PLoS One. 2017 Mar 10;12(3):e0173508. doi: 10.1371/journal.pone.0173508 (PMC5345810; doi:10.1371/journal.pone.0173508)
Supplement: S10 Table — (DOCX) [file pone.0173508.s010.docx]

**S10 Table: Upregulated genes in lung cancer**

| Name ID   \| pgsA \| Rv2612c \| \| --- \| --- \| \| Rv1744c \| Rv1744c \| \| Rv2416c \| Rv2416c \| \| ephB \| Rv1938 \| \| Rv3172c \| Rv3172c \| \| hypothetical protein \| ORF03310 \| \| PE family protein \| ORFD0295 \| \| Rv2843 \| Rv2843 \| \| Rv0093c \| Rv0093c \| \| Rv1170 \| Rv1170 \| \| Rv0387c \| Rv0387c \| \| Rv3572 \| Rv3572 \| \| hypothetical protein \| ORFD0320 \| \| Rv0397 \| Rv0397 \| \| gabD2 \| Rv0234c \| \| Rv1394c \| Rv1394c \| \| PE \| Rv0335c \| \| ilvB2 \| Rv3470c \| \| Rv2370c \| Rv2370c \| \| serA \| Rv2996c \| \| Rv3098c \| Rv3098c \| \| Rv2728c \| Rv2728c \| \| conserved hypothetical protein \| ORF01163 \| \| Rv3903c \| Rv3903c \| \| Rv3862c \| Rv3862c \| \| Rv1675c \| Rv1675c \| | Fold Change   \| 11.36593 \|  \| \| --- \| --- \| \| 3.488248 \|  \| \| 2.936101 \|  \| \| 2.671425 \|  \| \| 2.575722 \|  \| \| 2.557477 \|  \| \| 2.453452 \|  \| \| 2.430178 \|  \| \| 2.376449 \|  \| \| 2.307376 \|  \| \| 2.267977 \|  \| \| 2.236504 \|  \| \| 2.186576 \|  \| \| 2.163901 \|  \| \| 2.148965 \|  \| \| 2.143136 \|  \| \| 2.126548 \|  \| \| 2.106315 \|  \| \| 2.088341 \|  \| \| 2.079871 \|  \| \| 2.064331 \|  \| \| 2.064057 \|  \| \| 2.056293 \|  \| \| 2.048775 \|  \| \| 2.019116 \|  \| \| 2.013362 \|  \| |
| --- | --- | --- | --- | --- | --- | --- | --- | --- | --- | --- | --- | --- | --- | --- | --- | --- | --- | --- | --- | --- | --- | --- | --- | --- | --- | --- | --- | --- | --- | --- | --- | --- | --- | --- | --- | --- | --- | --- | --- | --- | --- | --- | --- | --- | --- | --- | --- | --- | --- | --- | --- | --- | --- | --- | --- | --- | --- | --- | --- | --- | --- | --- | --- | --- | --- | --- | --- | --- | --- | --- | --- | --- | --- | --- | --- | --- | --- | --- | --- | --- | --- | --- | --- | --- | --- | --- | --- | --- | --- | --- | --- | --- | --- | --- | --- | --- | --- | --- | --- | --- | --- | --- | --- | --- | --- |
